# Cost-effectiveness of peer role play and standardized patients in undergraduate communication training

**DOI:** 10.1186/s12909-015-0468-1

**Published:** 2015-10-24

**Authors:** Hans Martin Bosse, Martin Nickel, Sören Huwendiek, Jobst Hendrik Schultz, Christoph Nikendei

**Affiliations:** 1 Department of General Pediatrics, Neonatology and Pediatric Cardiology, University Children´s Hospital´, University Clinic Düsseldorf (UKD), Moorenstrasse 5, 40225 Duesseldorf, Germany; 2 Department of Anesthesiology, University of Heidelberg, Heidelberg, Germany; 3 Department of Assessment and Evaluation, Institute of Medical Education in Bern, Bern, Switzerland; 4 Department of General Internal Medicine and Psychosomatics, University of Heidelberg Medical Hospital, Heidelberg, Germany

## Abstract

**Background:**

The few studies directly comparing the methodological approach of peer role play (RP) and standardized patients (SP) for the delivery of communication skills all suggest that both methods are effective. In this study we calculated the costs of both methods (given comparable outcomes) and are the first to generate a differential cost-effectiveness analysis of both methods.

**Methods:**

Medical students in their prefinal year were randomly assigned to one of two groups receiving communication training in Pediatrics either with RP (*N* = 34) or 19 individually trained SP (*N* = 35). In an OSCE with standardized patients using the *Calgary-Cambridge Referenced Observation Guide* both groups achieved comparable high scores (results published). In this study, corresponding costs were assessed as man-hours resulting from hours of work of SP and tutors. A cost-effectiveness analysis was performed.

**Results:**

Cost-effectiveness analysis revealed a major advantage for RP as compared to SP (112 vs. 172 man hours; cost effectiveness ratio .74 vs. .45) at comparable performance levels after training with both methods.

**Conclusions:**

While both peer role play and training with standardized patients have their value in medical curricula, RP has a major advantage in terms of cost-effectiveness. This could be taken into account in future decisions.

## Background

Health systems worldwide are facing shortages in terms of resources, including their resources for medical education. Only few studies address the actual state regarding of allocation of resources within undergraduate medical education programs [1–3] with approximately 200 full-time equivalent (FTE) faculty members and 250 FTE residents for a program. There are little data to offer a valid base to estimate incremental costs (or savings) or revenue requirements. This lack of high-quality comparative studies with meaningful outcome measures allows no definitive conclusion regarding the general effectiveness of undergraduate curricula [4, 5].

Nonetheless, in light of such declining financial resources, curriculum designers are under increasing pressure to substantiate decisions for costly curriculum shares, amplified by the fact that – as Jones and Korn [5] point out – the present curricular innovations like small-group learning or investment in information technologies “*offer little solace to those concerned with mitigating the costs of medical student education”* anyway. The proposals to restructure undergraduate medical education in the name of cost-effectiveness to date largely turn out “*to be maneuvers to transfer […] costs to other entities*”; opportunities for significant reductions in the costs of medical student education are difficult to determine [5].

Given this dilemma, it is surprising how scarce literature is on what actually constitutes cost-effective education in healthcare [6]. In the field of health services, it seems inappropriate to monetize health effects as done in cost-benefit analysis (CBA). Hence, cost-effectiveness analysis (CEA) generally should be implemented. Here, costs are measured in monetary units, whereas benefits are accessed in outcome units: e.g., life years, severe adverse events averted. Our current literature search retrieved only 8 publications addressing cost-effectiveness and issues of medical education with the search terms (“cost effectiveness*”[TI] OR “cost benefit*”[TI]) AND analy* AND (“medical education” OR student* OR undergraduat*). Only four of these referred to the cost effectiveness or cost benefit of medical training itself [4, 6–8]. The costs within medical education programs seem worryingly high with – at least in part – little student satisfaction even in issues of perceived strong relevance (as shown for a course teaching on terminal care [7]). In an undergraduate program in dietetics the cost effectiveness of a “*coordinated course mode*” compared to a standard course was examined concluding that an improvement of curricular structure increases the course outcome (i.e., students passing an examination) at comparable expenses [8]. We found no high-quality comparative study providing sound evidence on cost effectiveness to decide which specific didactic method to favor for intended curricular outcomes.

Role-play as well as standardized patients play an important role in simulation based medical education (SBME) for procedural skills [9, 10] as well as communication training [11]. In SBME mainly validity of simulation, deliberate practice, and feedback seem to be relevant factors of success when it comes to practice [12]. Peer role play (RP) is a low-cost tool which is relatively easy to put into practice. RP allows switching of roles to experience both physician and patient perspectives. Through this experience of ambiguities in the communicational processes the trained communicating partners develop a better understanding of the involved physician-patient interaction dimensions [13, 14]. With carefully designed RP training sessions and well-trained tutors initial skepticisms towards RP may be resolved [15, 16]. It provides successful and targeted practice as well as useful feedback [14]. Nevertheless, RP needs careful planning “*because it is easy to use badly*” [13]. Standardized patient (SP) is an umbrella term both for a simulated patient, trained to simulate a patient’s illness, and an actual patient, trained to present their own illness, both in a standardized way [17, 18]. We refer to SP in this publication as simulated patients trained in a standardized way. SP are classified as low-technology instruments, but are expensive tools for training communication skills [19]. They provide a high degree of realism and have strong potential for training general and specific communication skills [10, 20, 21]. The key to SP’s success is their professional feedback [22, 23].

Regarding skills or communication training, there is a call for comparing the cost-effectiveness of (potentially more expensive) simulated learning experiences to lower fidelity interventions (as potentially less expensive) [24], but to our knowledge there are no data at all on assessing cost-effectiveness of specific methods in skills or communication training as such. The few studies that directly compare the use of role play and standardized patients (for a thorough review see Lane et al. [11] all agree that both methods can deliver comparable communication skills. Peer role play may even provide a methodological advantage in fostering a more empathic approach towards patients’ concerns. From an economical perspective, the cost of standardized patients is high: beyond the development of scenarios (necessary in both RP and SP-training), further costs for recruiting, coaching, expert case approval, contingency costs and operating costs arise. Role play is considered to be more economical but has yet to be quantified.

In a previous study, fifth year medical students were assigned to receiving a training in counseling parents of sick children either with RP (*N* = 34) or with SP (*N* = 35). Following the training, objective performance in parent-physician communication was assessed using the Calgary-Cambridge-Observation-Guide Checklist in a six-station OSCE. We were able to show that the training led to an increase in the post-intervention OSCE scores after RP and SP-training. This benefit was higher after RP than after SP-training due to superior performance in the domain *understanding of parents’ perspective* [24]. Based these outcomes, the present study to our knowledge is the first to reveal a cost-effectiveness analysis in the field of procedural training implemented with peer role play and with standardized patients.

## Methods

### Participants and training sessions

Fifth year medical students were randomly assigned to one of two groups receiving either communication training with peer role play (RP, *N* = 34) or standardized patients (SP, *N* = 35) within their pediatric rotation as described earlier [24]. Both groups were trained on the same nine cases over three training sessions addressing all predefined learning goals. Great care was taken to minimize bias by maintaining the same tutors, the same rotations in groups of three students allowing every student to take the active role of a physician once in every of the sessions, the same course materials, the same time for training and the same debriefing after the training for both groups. Only difference between the groups were SP taking the role of the patient in the SP group and the (supposedly) more professional feedback of the SP in the debriefing as compared to that of the peer previously in the patient’s role [24]. For more detailed information on the methodology of the underlying justification study we refer to our previous publication [24].

### Standardized patients

Data on standardized patients are stated in accordance with the recommendations of Howley et al. [25]. Overall, 19 individually trained standardized patients (*n* = 14 female, *n* = 5 male), with more than two years of experience at the Standardized Patients Training Centre of our Medical Faculty, were deployed. The standardized patients were used for case portrayal and providing oral feedback as well as for the objective structured clinical examination (OSCE); rating was performed by medical doctors.

### Assessment of effectiveness

**Fig. 1:**
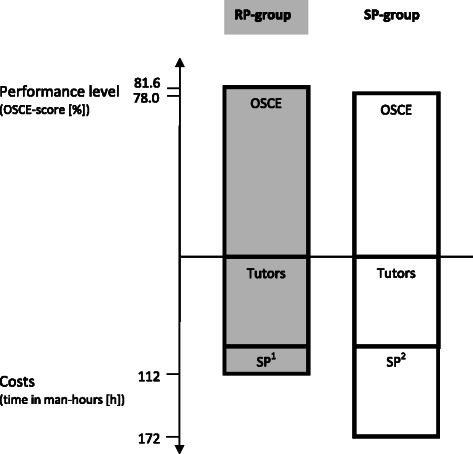
Cost-effectiveness analysis as ratio of effectiveness und man-hours. Cost-effectiveness analysis as ratio of effectiveness (OSCE score in %) und man-hours (in h) for the peer role play (RP)-group (*N* = 34; grey) and standardized patients (SP)-group (SP-group; *N* = 35; white). ^1^SP = standardized patients; for peer role play specific coaching of the SP for assessment (OSCE), and presence during the OSCE is taken into account. ^2^For SP-training, specific coaching of the SP for assessment (OSCE), communication training, and presence during the OSCE is taken into account

Outcome was assessed in an OSCE of six stations [24] addressing challenging parent-physician interactions with one standardized patient per station for both groups using the Calgary-Cambridge Referenced Observation Guide with global rating scales rated on visual analogue scales that range from 100 = completely agree to 1 = strongly disagree (see Fig. 1) [26].

### Assessment of costs

For the assessment of costs the total man-hours for coaching lecturers and standardized patients as well as for holding the training sessions were calculated by considering a) the specific coaching of standardized patients for their training cases and their presence in the training sessions (standardized patients-group), b) the coaching of the standardized patients for the final OSCE (both groups), as well as c) the presence of lecturers for the training sessions (both groups; Table 1).

**Table 1:** Costs for specific training

	Standardized patient-group (man-hours)	Peer role play-group (man-hours)
Standardized patients		
Specific coaching of standardized patients^a^	5	5
Specific training sessions	60	0
Assessment in the OSCE	21	21
Tutors		
Tutor training and preparation of sessions	5	5
Specific training sessions	60	60
Assessment in the OSCE	21	21
Total	172	112

^a^for communication training sessions as well as for employment in the OSCE

Contingency costs or costs for the general organization of the Standardized Patients Training Centre of our Medical Faculty were not incorporated. For the cost-effectiveness analysis a quotient of effectiveness (in %) and man-hours (in h) was determined.

### Ethical approval

The University of Heidelberg Ethics Committee waived requirements for an ethical approval procedure.

## Results

### Effectiveness

As shown in our prior study, both groups had a relatively high score in their assessment after the course with a significant difference in favor of training as peer role play (81.6 % ± 3.32 %; training with standardized patients 78.0 ± 6.23; p = .021 (see Fig. 1) [24].

### Costs

Costs for specific coaching of the standardized patients for communication training as well as for the OSCE were considered in both groups (5 man-hours) as well as the costs for employment of standardized patients for the OSCE (21 man-hours). The SP-group received a total of 60 man-hours of training. The costs of tutors were equal in both groups (training and preparation of sessions: 5 man-hours, training sessions: 60 man-hours and OSCE: 21 man-hours). Resulting costs in man-hours were 112 for the RP-group and 172 for the SP-group (+53.6 %).

### Cost-effectiveness analysis

In the cost-effectiveness analysis, a cost-effectiveness ratio of .74 for communication training with student role plays and a ratio of .45 for standardized patients were determined.

## Discussion

Both study groups, trained with peer role play or standardized patients, showed comparatively high scores in the post-intervention OSCE scores after their training as previously published [24]. This is in line with the sparse extent literature of comparative studies [11]. However, in our prior study, we found role play may even provide a methodological advantage in fostering a more empathic approach towards patients’ concerns [24]. In extant literature, the few previous comparative studies [11, 24] generally refer to the lower costs of peer role play in comparison to standardized patients in light of comparable effectiveness without, however, quantifying these. In the current study, we were able to show a clear quantified cost advantage of peer role play – as expected, but to our knowledge for the first time: peer role play incurred less costs than using standardized patients with a comparable effectiveness of both methods. The absolute costs were 53.6 % higher for training with standardized patients than for training with peer role play. In the cost-effectiveness analysis a better cost-effectiveness ratio was determined for peer role play (.74) compared to training with standardized patients (.45).

These significantly higher costs of standardized patients need to be weighed against their potential benefits. Standardized patients are extremely valuable both for training [14, 27] and assessment [28]. They appropriately and effectively interrupt peers – which in turn is significantly and positively associated with students’ grades making the simulated consultations an educational device with “*institutional power over the student*” [29]. Furthermore, the initial acceptance for standardized patients may be higher than for role playing [24] and the gateway hurdle may be lower with standardized patients [30]. This may specifically hold true for emotional demanding scenarios such as breaking bad news (unpublished observations of the authors).

Besides higher cost-effectiveness, peer role play offers the important advantage to gain patient insights – a fact that decisively might have contributed to our findings in our earlier study. This suggests that – if great care is taken – peer role play may not only be an equivalent educational device in many settings but leads to a better understanding of patient’s perspective and therefore seems to foster a more empathic approach towards patients’ concerns justifying its prominent role in medical curricula [24].

The immediate feedback following peer role play is of high didactic value [31] – and may be of comparable effect in both methods if feedback is structured and feedback givers are instructed carefully [24].

Given these considerations, there are several arguments to implement communication training programs with peer role play and as well with standardized patients. Nonetheless, our data show that standardized patients should be used thoughtfully as an educational device to minimize costs. Therefore it could be recommended that peer role play should be used to deliver communication skills in the early start of medical education as it seems to foster a more empathic approach towards patients’ concerns justifying its prominent role in medical curricula [24]. Standardized patients may make a very valuable contribution in more experienced medical students, when confronted with more demanding scenarios or scenarios that are difficult to be portrayed by peers and require emotional safety (i.e., breaking bad news), and for assessment [24, 25, 27].

## Limitations

We present an analysis of a communication training over a few weeks only – which may provide sustainable improvement in communication skills for a while [32]. But generally, a sustainable change in clinical practice would necessitate consolidation, i.e., further training [33] or continuous clinical supervision [34] which we didn’t provide. As we cannot judge sustainability we only determined the immediate costs of the trainings and cannot draw conclusions regarding cost-effectiveness of specific tools in communication training in the long run. In addition, it would be interesting to assess costs attributed to trainees-at-risk that would have benefited from a coaching – an argument that investing early on in expert communication interventions may result in significant overall savings later on.

## Conclusion

Both peer role play and training with standardized patients have their value in medical curricula. We demonstrate a major advantage regarding cost effectiveness for peer role play at comparable performance levels. Future decisions on the implementation of the two methods should take their costs into account. In our opinion, standardized patients should be reserved for training scenarios which are not suitable for peer role play, i.e., breaking bad news, or offer an option for trainee assessment.
